# “Top-down” and “bottom-up” strategies for wafer-scaled miniaturized gas sensors design and fabrication

**DOI:** 10.1038/s41378-020-0144-4

**Published:** 2020-05-04

**Authors:** Lin Liu, Yingyi Wang, Fuqin Sun, Yanbing Dai, Shuqi Wang, Yuanyuan Bai, Lianhui Li, Tie Li, Ting Zhang, Sujie Qin

**Affiliations:** 1 Department of Health and Environmental Sciences, Xi’an Jiaotong-Liverpool University, 111 Ren’ai Road, Suzhou, Jiangsu 215123 P. R. China; 2 Department of Environmental Science, University of Liverpool, Brownlow Hill, Liverpool, L69 7ZX UK; 3 i-Lab, Suzhou Institute of Nano-Tech and Nano-Bionics (SINANO), Chinese Academy of Sciences (CAS), 398 Ruoshui Road, Suzhou, Jiangsu 215123 P.R. China

**Keywords:** Sensors, Nanosensors

## Abstract

Manufacture of large-scale patterned nanomaterials via top-down techniques, such as printing and slurry coating, have been used for fabrication of miniaturized gas sensors. However, the reproducibility and uniformity of the sensors in wafer-scale fabrication are still a challenge. In this work, a “top-down” and “bottom-up” combined strategy was proposed to manufacture wafer-scaled miniaturized gas sensors with high-throughput by in-situ growth of Ni(OH)_2_ nanowalls at specific locations. First, the micro-hotplate based sensor chips were fabricated on a two-inch (2”) silicon wafer by micro-electro-mechanical-system (MEMS) fabrication techniques (“top-down” strategy). Then a template-guided controllable de-wetting method was used to assemble a porous thermoplastic elastomer (TPE) thin film with uniform micro-sized holes (relative standard deviation (RSD) of the size of micro-holes <3.5 %, *n* > 300), which serves as the patterned mask for in-situ growing Ni(OH)_2_ nanowalls at the micro-hole areas (“bottom-up” strategy). The obtained gas microsensors based on this strategy showed great reproducibility of electric properties (RSD < 0.8%, *n* = 8) and sensing response toward real-time H_2_S detection (RSD < 3.5%, *n* = 8).

## Gas sensors: Combining top-down and bottom-up

High-throughput and repeatable fabrication of miniaturized gas sensors is achieved by combining top-down and bottom-up techniques. Miniaturized gas sensors are typically manufactured by top-down techniques such as printing sensing materials on the sensor chips. However, achieving uniform and repeatable device performance is challenging. Here, a group led by Sujie Qin from Xi’an Jiaotong-Liverpool University and Ting Zhang from Suzhou Institute of Nano-tech, CAS combine top-down MEMS-based techniques with bottom-up in-situ growth of sensing materials to fabricate gas sensors. Specifically, MEMS based micro-hotplates are fabricated on a silicon wafer, followed by photolithography, then a de-wetting step assembles an elastomeric thin film with uniform micro-holes. Finally, in-situ growth of Ni(OH)_2_ nano-walls at the micro-holes creates the sensing device. The gas sensors showed reproducible electrical properties and sensing behavior for the detection of hydrogen sulfide gas.

## Introduction

High-throughput and low-cost wafer-scale fabrication of metal oxides based gas sensors with good reproducibility and uniformity are urgent demanded for wide applications in environmental monitoring^1,2^. Two main technological hurdles need to be overcome for low-cost wafer-scale fabrication, namely, how to efficiently form microscale-patterned sensing nanomaterials at specific locations on sensor chips and how to rationally integrate patterning methods with the micro-electro-mechanical-system (MEMS) technique (“top-down” microfabrication technique). During the past few decades, many advanced instruments and techniques have been utilized to obtain high-resolution material patterns on different substrates including printing^3,4^, precisely manipulated coating^5–7^, soft-photolithography-guided method^8–10^, controllable de-wetting^11–13^, and combined strategies of two or three techniques^14–16^. However, compared with in-situ growing method (“bottom-up” strategy), the interconnection between the sensing materials and the substrate is relatively weak and the obtained sensing material patterns are tend to be inhomogeneity, which can greatly influence the stability, uniformity, and reproducibility of sensors^17,18^.

A few studies have shown the feasibility to directly obtain patterned metal oxides on the sensor chips, such as laser-assisted method^19–21^, sputtering^22^ and self-lithographic chemical vapor deposition (CVD)^23–25^, which are compatible with MEMS fabrication techniques. However, for the laser-assisted method, continuous laser irradiation and complicated set-ups are required lead to increase the cost. For the sputtering method, it not only increases the cost but also leads to lower sensitivity based on plannar metal oxide films compared with three-dimension nanostructured metal oxides obtained via solution-based synthesis methods^22,26,27^. For self-lithographic CVD method, it is an appropriate yet relatively complicated method to fabricate micro-hotplate sensor arrays with different materials by applying different heating temperatures (300–800 °C) seperately^23–25^. Notably, template-guided controllable de-wetting method with the capability to precisely control the shape, size, and location of liquid droplets shows the feasibility to assemble uniform porous film due to the immiscibility of two different solutions^28^. Therefore, it is a potential approach to integrate “bottom-up” (in-situ growth) and “top-down” (MEMS fabrication techniques) strategies for wafer-scale fabrication of miniaturized gas sensors by utilizing the uniform porous film as a mask for in-situ growth of metal oxides. An ideal mask material requires good thermal stability and durability to acid and alkali solutions, and also must be easily removed. Although photoresists are common mask materials in the field of the MEMS, According to our previous work, thermoplastic elastomer (TPE) is an ideal cost-effective mask material due to its good chemical and thermal stability and can be easily peeled-off from the Si substrate^29,30^. In this work, we combined “bottom-up” (in-situ growth) and “top-down” (MEMS fabrication techniques) strategies for wafer-scale fabrication of miniaturized gas sensors with excellent reproducibility and high-throughput. Firstly, a “top-down” strategy was employed to fabricate the micro-hotplate wafer, which was followed by photolithography to form a dry film photoresist (DFP) dot array. The DPF dots serve as templates to assemble the porous TPE film with uniform micro-sized holes. In order to strengthen the interconnection between the macro-hotplate and the sensing materials, a “bottom-up” strategy was utilized to grow patterned NiO nanowalls at specific locations on the micro-hotplate wafer. The obtained miniaturized gas sensors showed excellent reproducibility of electrical properties (RSD < 0.8%, *n* = 8) and response toward H_2_S (RSD < 3.5%, *n* = 8).

## Results and discussion

**Fig. 1:**
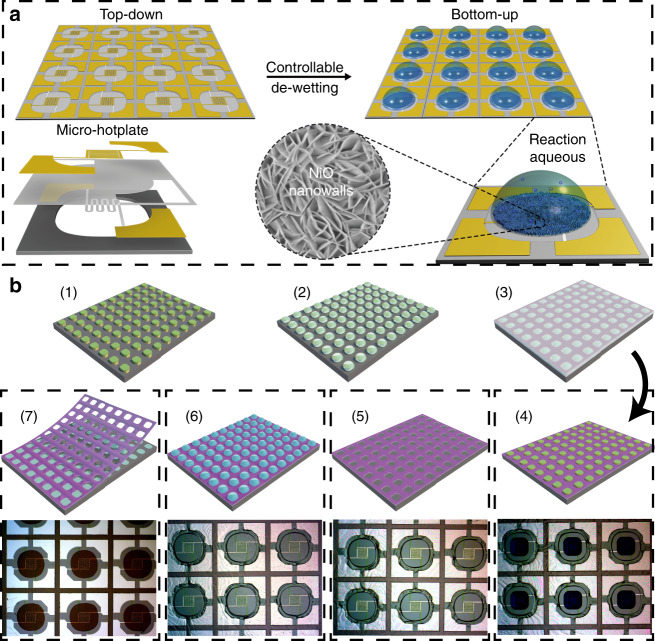
Schematic of fabrication of wafer-scaled gas sensors. **a** Schematic of a top-down and bottom-up combined strategy for fabricating wafer-scale miniaturized gas sensors; **b** schematic of template-guided controllable de-wetting and in-situ growth steps during wafer-scale fabrication process and relevant photograph results of some steps: (1) patterned dry film photoresist (DFP) dots were formed on the two-inch (2”) micro-hotplate wafer via photolithography; (2) the formed of water droplet array on the surface of DFP dots; (3) spin-coating of 18% TPE solution and waiting for evaporation of n-hexane; (4) the formed uniform porous TPE film with a gap to the DFP dots; (5) strip off of the DFP dots; (6) in-situ growth of Ni(OH)2 nanowalls; (7) peel off of the porous TPE film

The schematic structure of the micro-hotplate is shown in Fig. 1a, from bottom to top are silicon covered with a thin layer of silicon nitride (Si_3_N_4_) on both sides of silicon, heating electrode (Pt, ~200 nm), SiO_2_ (insulator layer), and the working electrode (Pt, ~200 nm), respectively. After constructing all of the structures (including heating electrode, insulator layer, and working electrode) on the front side, reactive ion etching (RIE) and silicon deep etching were employed to etch the back of the silicon (etching area: 720 μm × 720 μm; etching depth: 250 μm). In addition, a wet-etching method was used to further etch the silicon on the back side in 25% tetramethylammonium hydroxide (TMAH) solution at 90 °C with a specially designed fixture (Fig. S1b) to protect the front side. The size of each micro-hotplate was 1.2 mm × 1.2 mm. Fig. S2 shows the schematic graph of micro-hotplate after growth of NiO nanowalls to illustrate components of the micro-hotplate. As the micro-hotplate becomes very fragile after the wet-etching process, the wet-etching was not carried out until the in-situ growth of the Ni(OH)_2_ nanowalls in this case. A controllable template-guided de-wetting method was utilized as a bridge to integrate “top-down” and “bottom-up” strategies to implement in-situ growth of Ni(OH)_2_ nanowalls at specific locations for wafer-scale fabrication (Fig. 1a). The details of assembling porous TPE films are demonstrated in Fig. 1b. Firstly, the DFP dots were formed on the two-inch (2”) micro-hotplate wafer via photolithography (Fig. 1b (1)). Then, water was spin-coated onto the micro-hotplate wafer and water droplets were pinned by the DFP dots during the template-guided controllable de-wetting process (Fig. 1b (2)). Subsequently, TPE solution (18 wt%, in *n*-hexane) was spin-coated (Fig. 1b (3)). After the evaporation of hexane, ethanol was spin-coated two times to release the water to obtain a uniform porous TPE film as displayed in Fig. 1b (4). Because the ethanol can dissolve the ultrathin TPE film which is covering on the surface of water droplets. In the optical picture result of step ‘b (4)’, an obvious gap between the DFP dots and the porous TPF film was formed. Because the water droplet is immiscible with the hexane and the density of water is greater than that of hexane, and then the TPE solution can be effectively isolated from the DFP dots. Since the DFP dots will absorb water and swell after immersing into a 5% TMAH solution, the formed gap can facilitate striping off the DFP dots by offering enough swelling space (Fig. 1b (5)). Without the gap, the swelled DFP dots would tightly contact with the TPE film and the TPE film would peel off from the substrate with the DFP dots together during the striping off process. After assembly of the porous TPE film, the micro-hotplate wafer covered with the porous TPE film was immersed in the mixed solution of 37.5 mM NiCl_2_, 200 mM NH_4_Cl, and 68.75 mM NaOH to in-situ grow the Ni(OH)_2_ nanowalls at micro-hole areas (Fig. 1b (6))^31^. The photograph of step ‘b (6)’ displays the micro-hotplates covered with the porous TPE film after growth of the Ni(OH)_2_ nanowalls. The porous TPE film was peeled off effortlessly and thoroughly with the assistance of ethanol without contaminating the Ni(OH)_2_ nanowalls (Fig. 1b (7)). The optical photograph of the step ‘b (7)’ shows the micro-hotplates covered with the NiO nanowalls after removing the porous TPE film and annealing at 400 °C for 2 h under an argon atmosphere.

**Fig. 2:**
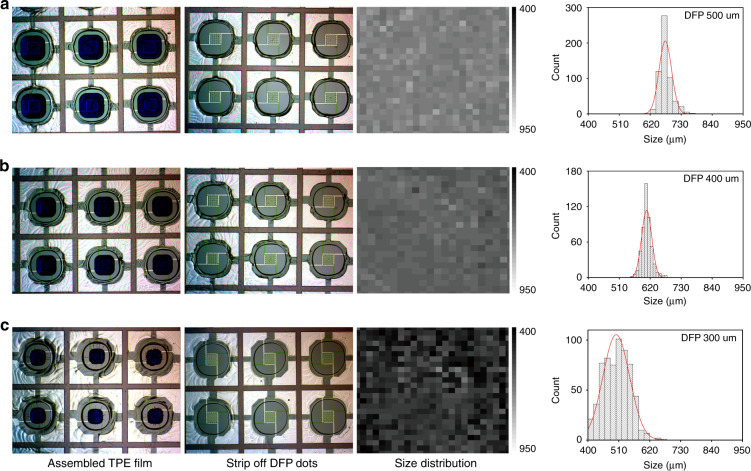
Size distribution of the micro-holes. **a**–**c** The photographs of porous TPE films and the size distribution of micro-holes based on different sizes of DFP dots. From left to right of each row are the picture of assembled TPE film on the micro-hotplates surrounding the DFP dots, the picture of porous TPE film after stripping off the DFP dots, the size distribution mapping and histogram graphs of size of the micro-holes, respectively

The reproducibility of sensors is significantly dependent on the size uniformity of micro-holes of the porous TPE film. The size of micro-holes is defined by measuring the distance from center point of one edge to the center point of the opposite edge and calculating the average value. The porous TPE films with different size of micro-holes were obtained by varying the size of DFP dots (from 500 μm to 300 μm; Fig. 2). Obviously, the size of micro-holes decreased along with decrease of the size of DFP dots. From the histogram graph of size distribution of micro-holes, it can be seen the size of micro-holes decreases from ~763.69 ± 22.32 μm to ~500.87 ± 47.97 μm. The size dispersion of the obtained micro-holes based on different size of the DFP dots is shown in Table 1. According to Table 1, the relative standard deviation (RSD) values (*n* > 300) of the size of micro-holes based on 500 μm, 400 μm, and 300 μm DFP dots are 3.31%, 2.94%, and 9.58%, respectively. For the DFP dots larger than 300 μm, the size distribution of micro-holes is significantly narrowed (RSD < 3.5%) indicating that the obtained micro-holes of the porous TPE films have an excellent uniformity which is a precondition to ensure the reproducibility of gas sensors.

**Table 1:** The size dispersion of micro-holes based on different size of DFP dots.

DFP Size (μm)	*N*	SD (μm)	Mean (μm)	RSD (%)
500	576	22.32	673.69	3.31
400	510	17.85	607.49	2.94
300	575	47.97	500.87	9.58

Working temperature distribution is another important parameter that can influence the reproducibility and needs to be taken into consideration since the performance of the gas sensors (sensitivity, response, and recovery speed) are considerably depending on the working temperature. The working temperature is determined by applying a certain potential on the Pt heating electrode due to Joule heating. As the heating area (300 μm × 300 μm) is limited by the serpentine Pt electrode at the center of the micro-hotplate, a temperature gradient forms from the center to the edge of the micro-hotplate according to the COMSOL simulation result of the temperature distribution (Fig. S3). Therefore, in order to diminish the temperature differential on the sensing materials, a smaller area of the sensing materials is better. In addition, as the lowest RSD value (2.94%) of macro-holes is obtained based on 400 μm DFP dots, DFP dots (400 μm) were chosen to assemble porous TPE film to guarantee the reproducibility of the obtained sensors.

**Fig. 3:**
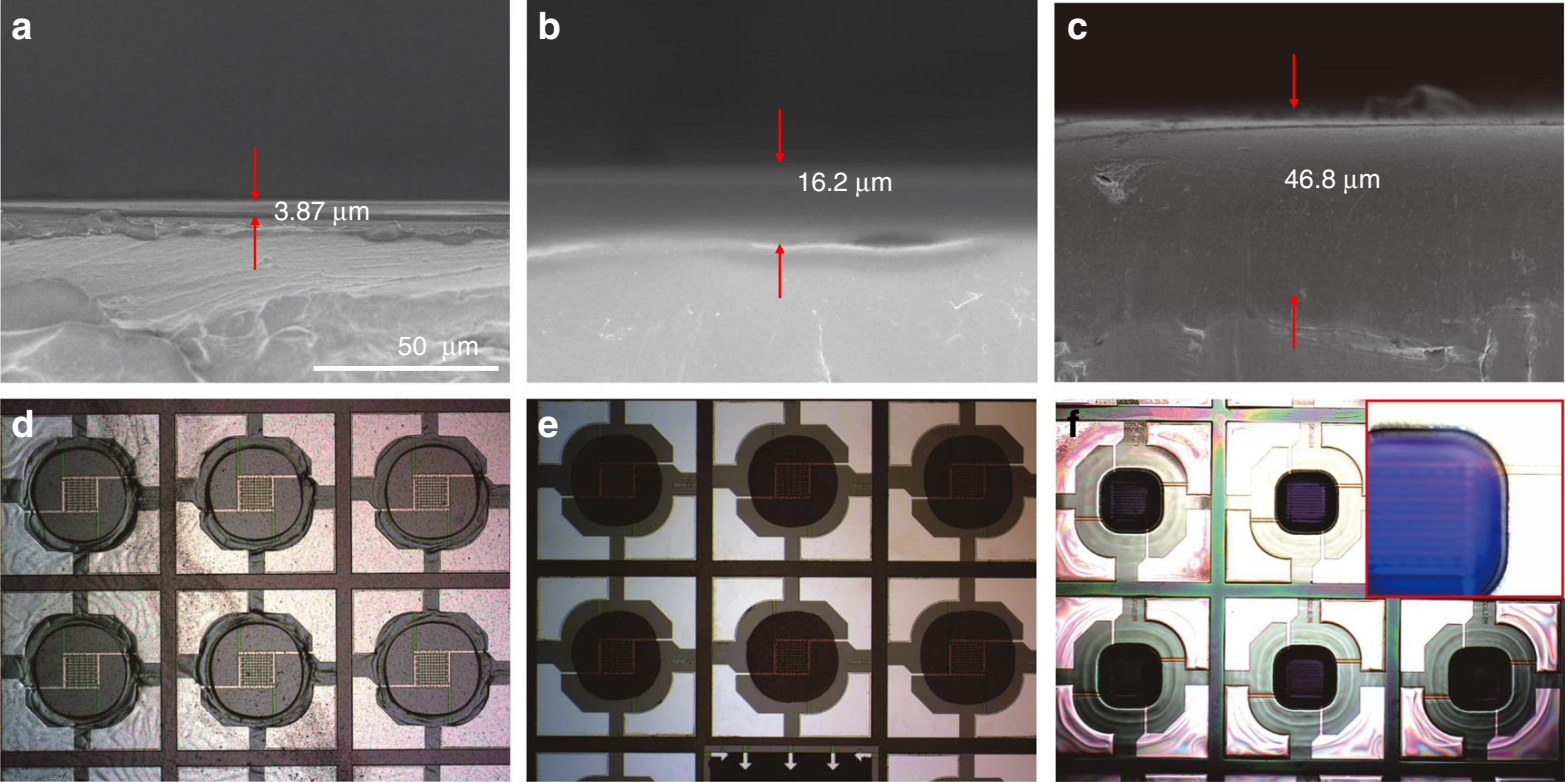
Effect of the concentration of the TPE solution. Sross-sectional SEM images of TPE film on polyethylene terephthalate (PET) substrate obtained from different concentration of TPE solution: **a** 10%; **b** 18%; **c** 25%; **d** the peel off result of the porous TPE film (10%) after in-situ growth of Ni(OH)_2_ nanowalls; **e** the peel off result of the porous TPE film (10%) after in-situ growth of Ni(OH)_2_ nanowalls and annealed at 400°C for 2 h; **f** the photograph of spin-coated TPE film (25%) after spin-coating ethanol

Based on the schematic fabrication process, the assembled porous TPE film should be thoroughly peeled off after in-situ growth of the Ni(OH)_2_ nanowalls. Thus, the effect of thickness of the TPE film was studied by assembling the porous TPE films with different concentrations of TPE solution (10, 18, and 25 wt%). The thicknesses of the TPE film increased from 3.84 μm to 46.8 μm when increasing the concentration of TPE solution owing to the increased viscosity (Fig. 3a–c). Figure 3d, e reveal the peeling-off results of the TPE film based on 10 and 18% TPE solutions, obviously the thinner one obtained from the 10% TPE solution was failed to be peeled off from the micro-hotplate wafer due to the enhanced interconnection between the TPE film and the substrate after growth of the Ni(OH)_2_ nanowalls. When using the 25% TPE solution, it was failed to obtain the porous TPE film due to the increased thickness of the TPE film compared with the TPE film obtained by 18% TPE solution. Although spin-coating ethanol can dissolve the ultrathin TPE film to release water droplets (Fig. 3d, e), which is covered on the surface of water droplets, the solubility of the thick TPE film in ethanol is rather limited (Fig. 3f)^29^. So even the water droplets can insulate the DFP dots from the TPE film (inset picture in Fig. 3f), the TPE film still remained and covered on the surface of the water droplets after spin-coating ethanol (Fig. 3f). Therefore, the 18% TPE solution was chosen to assemble porous TPE film.

**Fig. 4:**
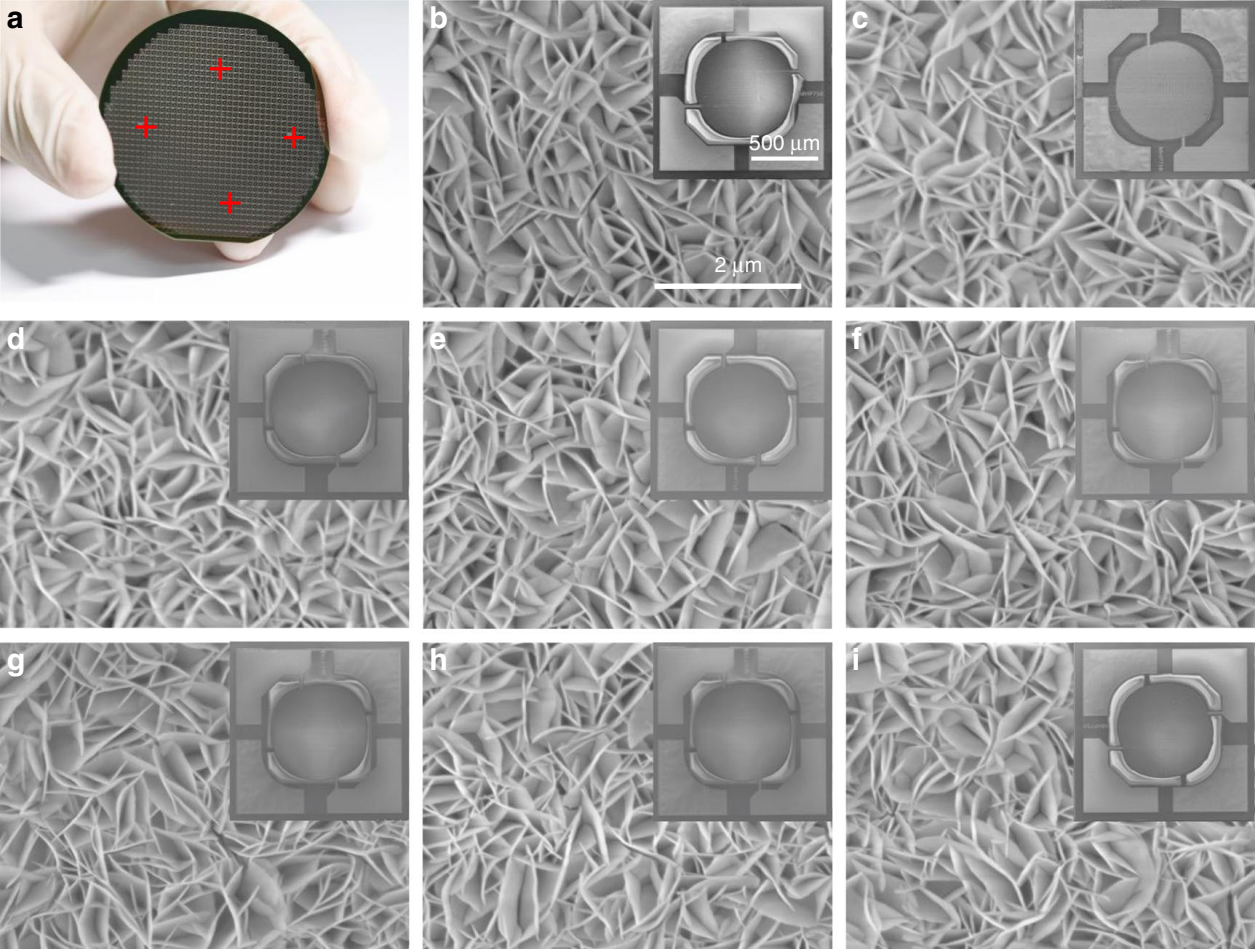
SEM characterization of selected sensors. **a** The optical picture of wafer-scale micro-hotplate; **b**–**j** SEM images of 8 sensors which are randomly chosen in the marked area of the wafer-scale micro-hotplate. Scale bar of sensor chip is 500 μm and for NiO nanowalls is 2 μm

Crystal structures of sensing materials on the silicon substrate before and after annealing treatment were further verified by X-ray diffraction (XRD) (Fig. S4). All of the reflection peaks are consistent with hexagonal crystal phase of β–Ni(OH)_2_ (JCPDS file NO.14-0117). For the XRD pattern of NiO nanowalls, the diffraction index peaks correspond with standard values (JCPDS file NO. 47-1049), indicating that the in-situ growth of Ni(OH)_2_ nanowalls are completely converted to NiO nanowalls. The unexpected peak located at 69° is ascribing to the diffraction peak of silicon substrate. Eight gas sensors were randomly picked out from the four labeled areas on the micro-hotplate wafer and characterized by scanning electron microscopy (SEM; Fig. 4 and Fig. S5) after annealing treatment and wet-etching process. These images reveal that NiO nanowalls are oriented vertically growing at the center of the micro-hotplate and exhibit uniform morphology without obvious cracks either in overall view of sensor or in local zoom images of the NiO nanowalls.

**Fig. 5:**
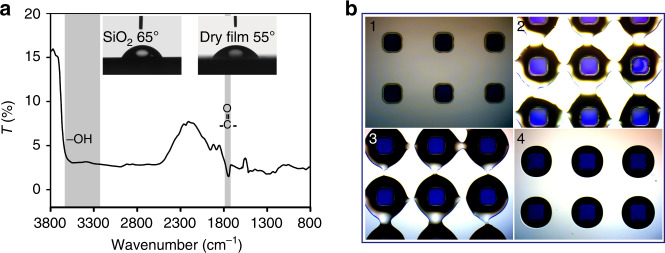
De-wetting mechanism and de-wetting process. **a** Fourier transform infrared spectra (FTIR) result of DFP after Standard UV exposure (10s) and contact angle results of SiO_2_ and DFP (inset pictures); **b** a series of optical images of process (from stage 1 to stage 4) of forming water droplets based on template-guided controllable de-wetting method using 400 μm DFP dots

**Fig. 6:**
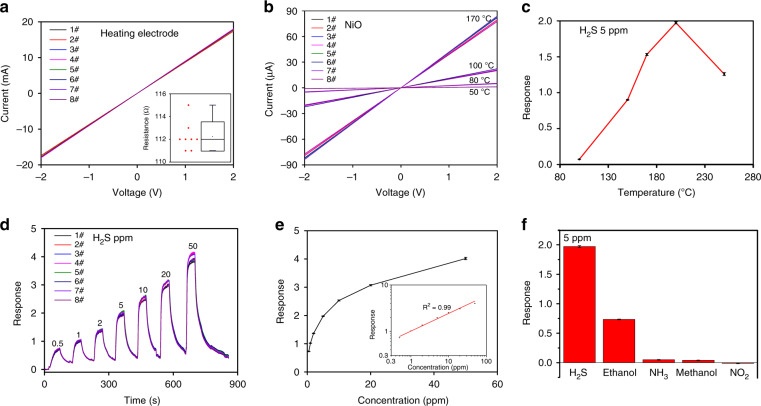
Electric and gas sensing performance test. **a** I–V curves of heating electrodes of eight gas sensors from −2V to 2V (inset graph is the box chart of resistance of heating electrodes); **b** I–V curves of eight sensors at different temperatures; **c** working temperatures dependent response of eight gas sensors to 5ppm H_2_S; **d** dynamic response of eight gas sensors to different H_2_S concentrations; **e** scatter graph of response to various H_2_S concentrations (inset graph is the linear relationship of logarithm of response with the logarithm of H_2_S concentrations); **f** response of eight gas sensors toward different gases and the concentration is 5 ppm

In order to further investigate the assembly mechanism of the water droplet array based on template-guided method, a piece of DFP film was characterized with Fourier transform infrared (FTIR) spectroscopy and water contact angle after exposure to standard ultraviolet (UV) (Fig. 5a). As the DFP mainly consists of epoxy- or acrylic-based composites, it contains abundant carboxyl and hydroxyl groups^32,33^. The FTIR data of the DFP film also confirms this result, which shows a very strong broad absorption peak (around 3200 cm^−1^ to 3650 cm^−1^) and a narrow peak (around 1740 cm^−1^) assigned to hydroxyl and carboxyl groups, respectively. Those function groups guarantee the DFP has a better hydrophilicity compared with SiO_2_ which is further confirmed by the water contact angle results (Fig. 5a). This hydrophilic property ensures that the DFP dots can efficiently pin water during the template-guided controllable de-wetting process. The specific process of assembling water droplets based on 400 μm DFP dots (Fig. 5b) has four different stages: (1) before spin-coating water; (2) ruptures appear at the water film; (3) contact lines gradually recede according to the contour of the DFP dots; (4) the water film completely breaks into discrete water droplets to cover on the surface of DFP dots. Obviously, ruptures appear at the central point of diagonal of DFP dots because the water film preferentially ruptures at the thinnest locations during the de-wetting process^12,13^. The de-wetting is a very common phenomenon in nature that liquid films break into hemi-spherical cap droplets and remain where there are surface defects, and artificially-induced defects. These defects can provide nucleation sites (e.g., patterned microstructure) during the de-wetting process due to a heterogeneous mechanism^11–13,34,35^. Hence, the patterned DFP dots will not only work as pinners to pin water, but also serve as the nucleation sites. Similar phenomena can be observed by varying the size of the DFP dots (Fig. S6). The size, shape, and location of the water droplets are determined by the DFP dots and the DFP dots can work as a fixer to ensure that the anchored water droplets will not shift to other locations when spin-coating TPE solution. As the periodicity (λst) of the DFP dots is constant (1200 μm) and is identical to the length of micro-hotplate, the distance between the edge of adjacent DFP dots (ld) varies when tuning the DFP dot size. Thus, the de-wetting process becomes progressively slower by increasing the DFP dot size due to the enhanced capillary force between the adjacent DFP dots, which is resulted from the decrease of ld. When the DFP dot size decreasing to 300 μm, it is not easy to precisely control the time of drop casting and spin-coating of the TPE solution (within 1–2 s), because the de-wetting process is too fast and leads to a relatively poor uniformity of the micro-holes (Fig. 2). Eight gas sensors were randomly picked out from the wafer and wire-bonded for testing. The reproducibility and uniformity of electrical and sensing performances of the 8 sensors were presented in Fig. 6. The I–V curves of the heating electrode are almost overlapping (Fig. 6a) indicating that the fabricated micro-hotplates possess great uniformity and reproducibility with a very small RSD value (<0.8%). Fig. 6b demonstrates the I–V curves of the 8 sensors at different heating voltages after in-situ growth of patterned NiO nanowalls at the center of micro-hotplates. The resistance of NiO nanowalls decreases with increasing heating voltage due to their semiconducting property. The RSD values of the eight gas sensors under different temperatures (40–170 °C) were 3.4%, 2.9%, 3.6%, and 2.9%, respectively, demonstrating good electrical uniformity. Figure 6c illustrates the sensing responses of the selected sensors toward 5 ppm H_2_S by varying the working temperatures (100–250 °C). Evidently, the response of the 8 sensors sharply increased by increasing the temperature and reached a maximum value at 200 °C, and then steeply declined. Therefore, all other measurements were made at 200 °C. Figure 6d shows the dynamic response of the sensors to different concentrations of H_2_S. Obviously, all sensors also demonstrate high sensing performance uniformity and reproducibility with a small RSD (<5%). The relationship of response toward H_2_S concentrations is displayed in Fig. 6e. The response rapidly increases at low H_2_S concentrations (<10 ppm) and then become slower with further increasing H_2_S concentrations as adsorption of H_2_S gradually reaches saturation according to Langmuir adsorption theory. However, there is a good relationship between the response in logarithm scale and H_2_S concentrations (*R*^2^ = 0.99, inset graph of Fig. 6e). The response of the gas sensors to different gases is illustrated in Fig. 6f. All the gas sensors can selectively detect H_2_S. The sensing mechanism of NiO nanowalls based sensor toward H_2_S may be described as follows. NiO as a p-type metal oxide semiconductor, in which containing many oxygen vacancies. When the sensor stays in air, O_2_ molecules will adsorb onto the surface of NiO nanowalls and extract electrons from Ni^2+^ to form ionized oxygen species such as O^2−^, O_2_^−^, and O^−^ and the Ni^2+^ is oxidized to the Ni^3+^ ^36,37^. Thus, the resistance of the sensor is low in the air attributing to the increased hole concentration of NiO nanowalls. When injecting H_2_S, the H_2_S molecules will react with the ionized oxygen species and donate trapped electrons back to NiO nanowalls causing the decreasing of hole concentration. Finally, the resistance of the sensor increases. The sensing response of different gases under different working temperatures was studied (Fig. S7). When the working temperature is around 100 °C, the sensor shows the highest sensing response to NO_2_ (<0.2) against other gases. By gradually increasing the working temperatures (150, 170 °C), the sensing response of the sensor to H_2_S dramatically increased against other gases and reached the highest response value when the working temperature is 200 °C (Fig. S7 and Fig. 6c). By further increasing the temperature to 250 °C, the sensing response of sensor to ethanol greatly increased. Thus it is difficult to distinguish H_2_S from ethanol at 250 °C. The selectivity toward H_2_S (Fig. 6f) is possibly ascribing to low bond dissociation energy of H-S compared with other tested interference gases, it means that H-S can be easily broken and oxidized to form SO_2_^38,39^.

## Materials and methods

### Chemicals

All reagents are analytical grade and are used without further purification. *n*-Hexane, sodium carbonate (Na_2_CO_3_), ethanol, Ni(OH)_2_, NaOH and NH_4_Cl were purchased from Sinopharm Chemical Reagent Co., Ltd. and 25% tetramethylammonium hydroxide (TMAH) was purchased from Jianghua Microelectronics Materials Co., Ltd. TPE (C6612T-A78) was obtained from GainShine Co., Ltd. and DFP (SAF2100) was a negative-type DFP bought from Dupont Co., Ltd. De-ionized (DI) water was obtained from a Millipore system (resistivity 18.2 MΩ.cm). TPE was dissolved in *n*-hexane through magnetic stirring and ready for use. The 2-inch silicon wafers (300 μm, double sides polished, Tianjing Yunjing Technology Co., Ltd.). Micro-hotplate wafers were fabricated by Leanstar-Tech Co., Ltd., which contain more than 1500 micro-hotplates in each 2-inch wafer.

### Preparing porous TPE film

The DFP film was cut to fit the size of silicon wafer and then laminated on the prepared micro-hotplate wafer at a specific temperature (90 °C) and baked at 60 °C for 15 min. Standard UV exposure (10 s) was performed at 365 nm to obtain patterned DFP dots via photolithography. After treatment in the developing solution (2% Na_2_CO_3_) for 10 min at room temperature, the unexposed area of DFP film was thoroughly removed. Then, water was spin-coated (500 rpm, 9 s) on the micro-hotplate wafer to form uniform water droplets via the template-guided controllable de-wetting process. Subsequently, 18% TPE solution was spin-coated (500 rpm, 9 s) on the wafer and this was followed by spin-coating ethanol after the evaporation of *n*-hexane ( ~5 s). 5% TMAH solution was applied to strip off DFP dots and then rinsed with water and ethanol for few times. Finally, the micro-hotplate wafer covered with porous TPE film was obtained and ready for growth in situ of Ni(OH)_2_ nanowalls.

### Characterization

XRD (Bruker AXS, D8 Advance) and SEM (Hitachi-s4800) were used to analyse the chemical composition and morphology of synthesized materials. I–V curves were measures with Agilent B1500A. Functional groups of DFP was tested by FTIR (Nicolet iN10). The water contact angle (WCA) measurements were conducted on an OCA20 machine (Data-Physics, Germany) at ambient temperature. Optical images and videos were captured by using a Canon Camera (Nikon, Japan) and microscope (Motic SMZ-168) which is connected with CCD camera.

### Gas sensing

After growing the Ni(OH)_2_ nanowalls, the porous TPE film was peeled off from the micro-hotplate wafer. Then, the micro-hotplate wafer was placed in the fixture (Fig. S1b) by exposing the back of micro-hotplate and then immersed in 25% TMAH at 90 °C in an oil bath to further remove the silicon at the back side of the micro-hotplate. After annealing treatment (Ar, 400 °C, 2 h), the micro-hotplates with sensing materials (NiO nanowalls) were wire-bonded to the test substrate. The performance of the sensors was measured simultaneously in a WS-30A static analysis system (Hanwei Electronics Co. Ltd., Henan Province, China) with the constant loop voltage of 5 V. Typically, the sensors were placed in a gas chamber (18 L) in air. The different concentrations of target gas (ethanol and methanol) was obtained by injecting a certain amount target solution into the chamber. The precise concentration was calculated according to following formula^40^:

$${{C}} = \frac{{22.4 \times \varphi \times \rho \times V1}}{{M \times V2}}$$

where *C* (ppm) is concentration of target gas, *φ* is volume fraction of target gas, ρ (g/mL), *V*1 (μL) and *M* (g/mol) are density, injected volume and molecular weight of target solution, respectively and *V*2 (L) is volume of chamber. For the target gases such as ammonia (NH_3_), toluene (C_7_H_8_), nitrogen dioxide (NO_2_) and hydrogen sulfide (H_2_S), the different concentrations were implemented by simply diluting the high concentration of gas sources. The sensing response of sensor is defined as Δ*V*/*V*g, where Δ*V* is the absolute differential of voltage on sensor in air and target gas, *V*g is the voltage on sensor in target gas. All the sensing measurements were performed under laboratory conditions (RH: 50–60%; temperature: 20–25 °C).

## Conclusion

“Top-down” and “bottom-up” strategies were combined to fabricate wafer-scale miniaturized gas sensors. “Top-down” strategy (MEMS fabrication techniques) guarantees the fabricated micro-hotplates with great reproducibility for further in-situ growth of Ni(OH)_2_ nanowalls. The template-guided controllable de-wetting method was utilized to integrate “top-down” and “bottom-up” strategy by assembling the uniform porous TPE film to serve as the mask for growing Ni(OH)_2_ nanowalls. In order to obtain porous TPE film with uniform micro-holes, the effects of the size of DFP dots and the concentration of TPE solution were systematically studied. Thus, a 400 μm DFP dots and 18% TPE solution were selected to assemble porous TPE film (RSD of the size of micro-holes <3.5%). The reproducibility and uniformity of obtained gas sensors were studied by randomly choosing 8 gas sensors at different sites via various methods such as SEM, I–V curves of heating electrodes and also the NiO nanowall, and dynamic gas response. Notably, the obtained gas microsensors showed great reproducibility of electrical properties (RSD < 0.8%, *n* = 8) and response toward real-time H_2_S detection (RSD < 3.5%, *n* = 8).This fabrication strategy shows great potential to fabricate wafer-scaled miniaturized gas sensors based on different sensing materials due to the good chemical and thermal stability of TPE film. The template-guided controllable de-wetting method also shows the feasibility to assemble different porous films and patterned materials.

## Supplementary information

“Top-down” & “Bottom-up” Strategies for Wafer-scaled Miniaturized Gas Sensors Design and Fabrication
