# Supplementary material for: “Top-down” and “bottom-up” strategies for wafer-scaled miniaturized gas sensors design and fabrication
Source: Microsyst Nanoeng. 2020 May 4;6:31. doi: 10.1038/s41378-020-0144-4 (PMC8433434; doi:10.1038/s41378-020-0144-4)
Supplement: Supplementary file 1 — “Top-down” & “Bottom-up” Strategies for Wafer-scaled Miniaturized Gas Sensors Design and Fabrication [file 41378_2020_144_MOESM1_ESM.docx]

Supporting Information

“Top-down” & “Bottom-up” Strategies for Wafer-scaled Miniaturized Gas Sensors Design and Fabrication

Lin Liu, Yingyi Wang, Fuqin Sun, Yanbing Dai, Shuqi Wang, Yuanyuan Bai, Lianhui Li, Tie Li, Ting Zhang*, and Sujie Qin*


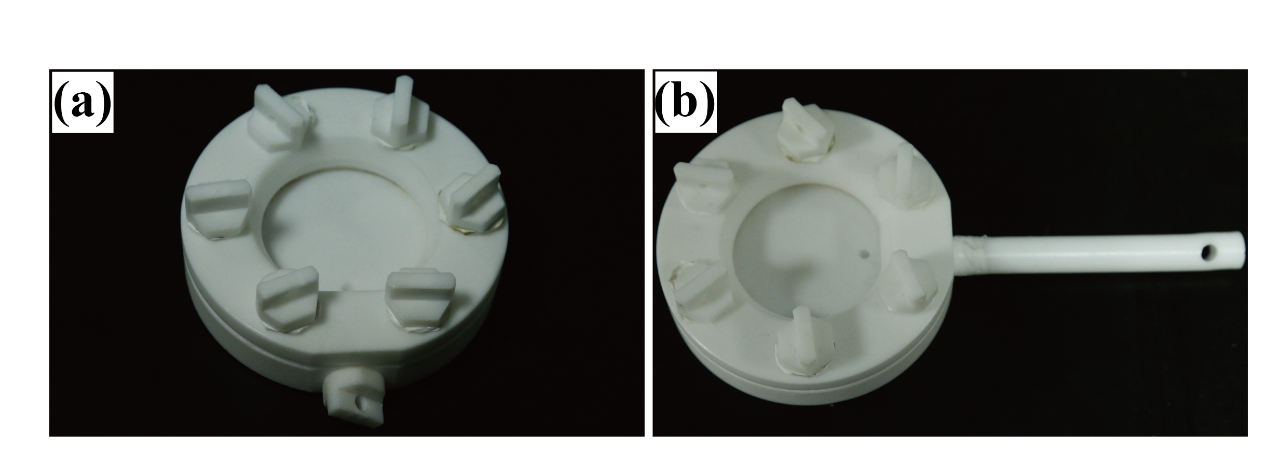


Figure S1. pictures of a designed fixture (a) is used for *in-situ* growth of Ni(OH)_2_ nanowalls; (b) is used for wet-etching of silicon on the back of micro-hotplate wafer.


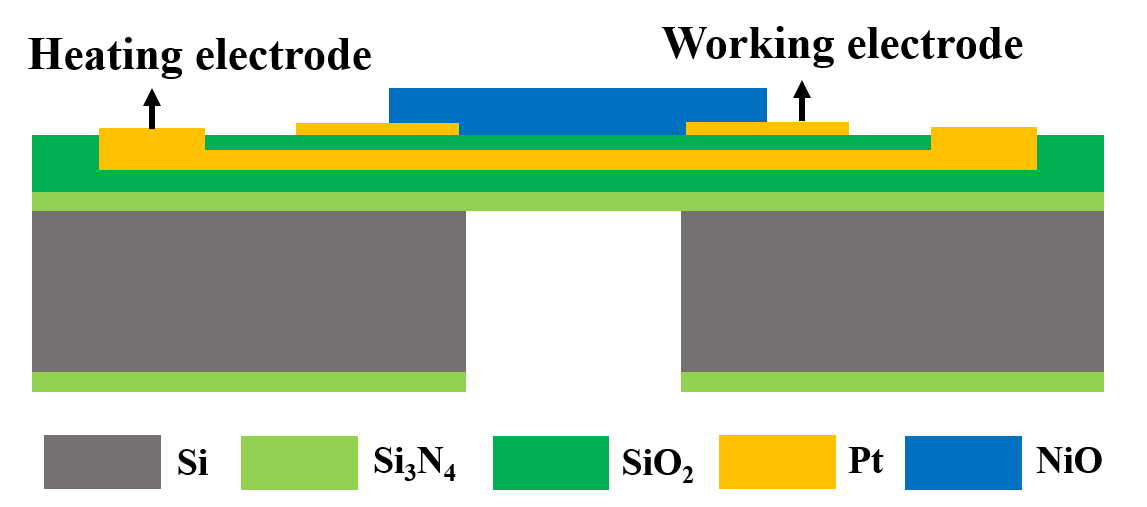


Figure S2. Schematic profile graph of the micro-hotplate after growth of NiO nanowalls.


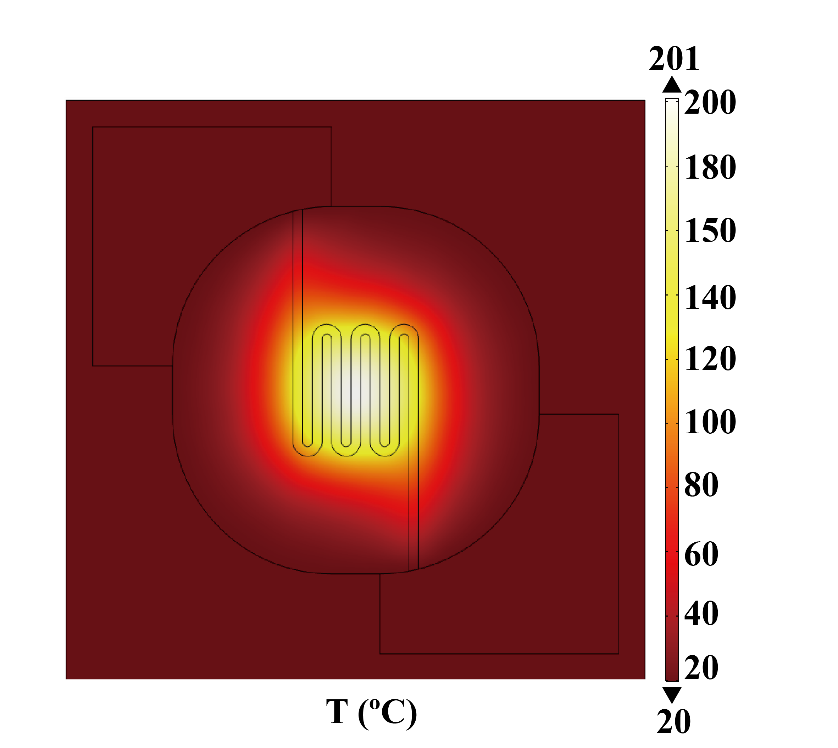


Figure S3. Temperature distribution simulation result on micro-hotplate.


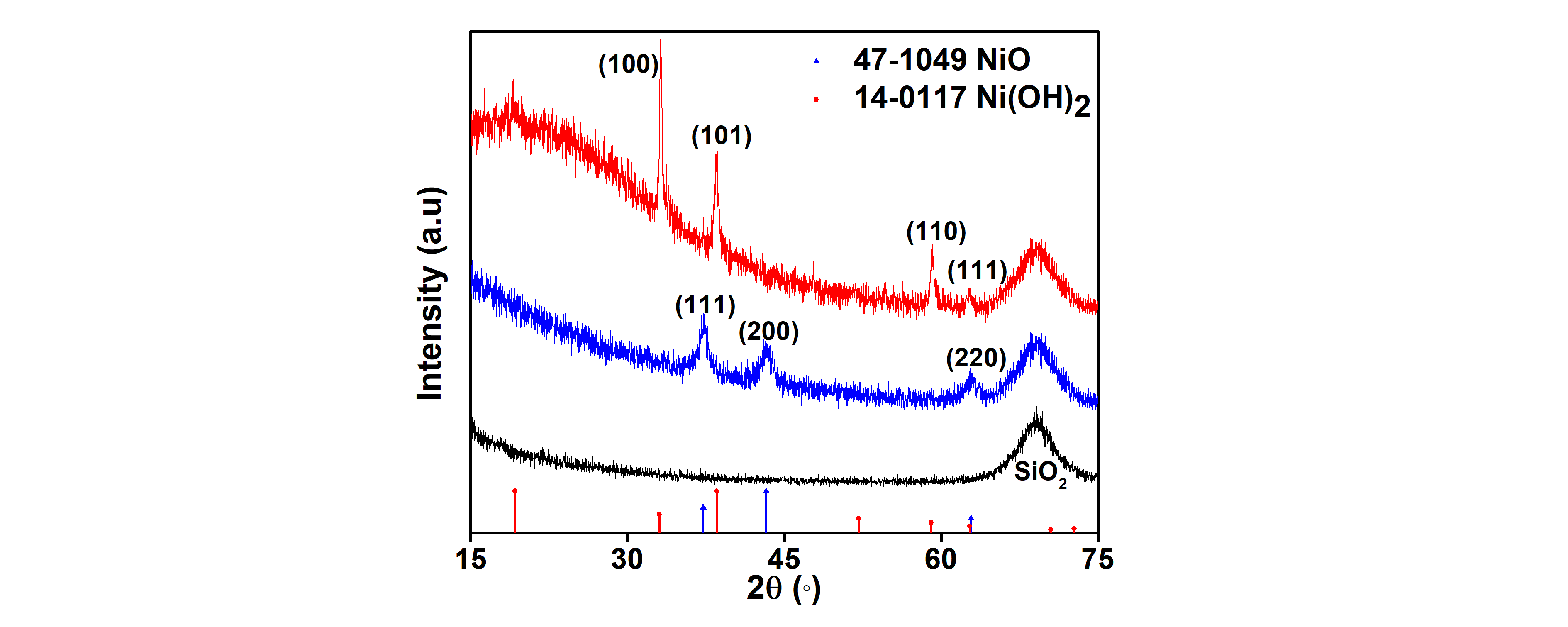


Figure S4. SEM images of 4 sensors which are randomly picked in different areas of wafer. Scale bar of sensor chip is 500 μm and for NiO nanowalls is 5 μm.


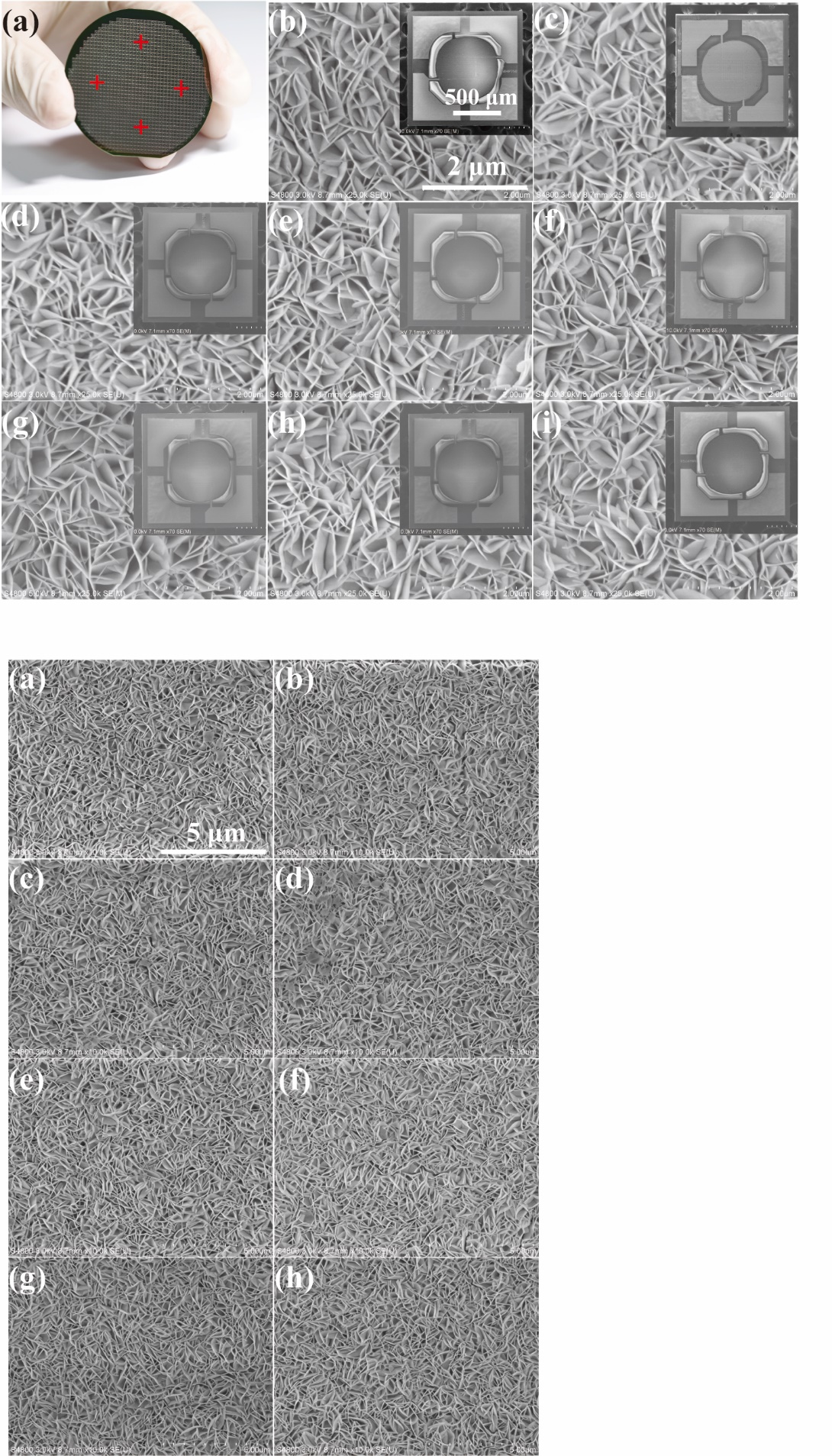


Figure S5. SEM images of 8 sensors which are randomly chosen in the marked area of the wafer-scale micro-hotplate (scale bar: 5 μm).


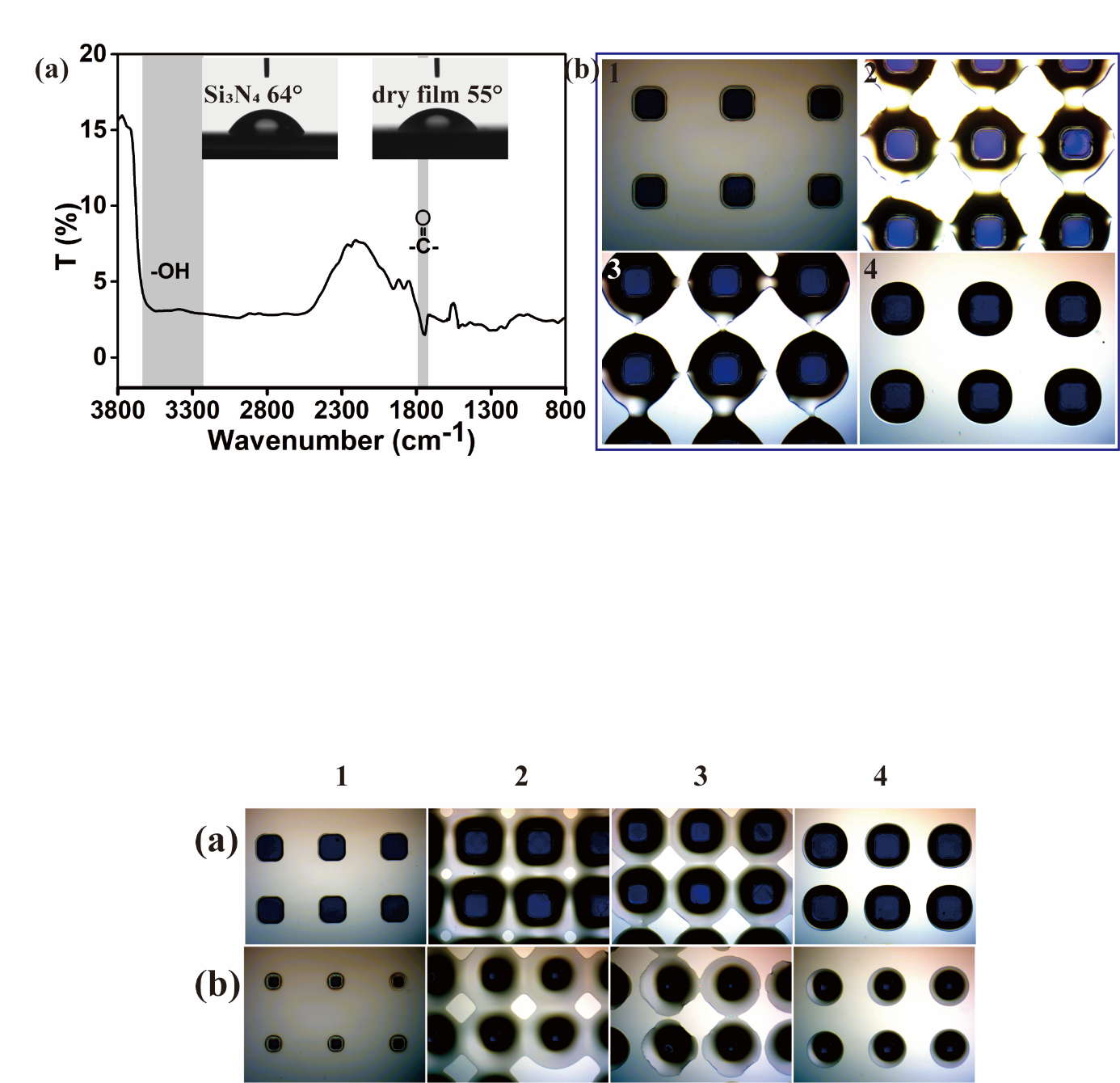


Figure S6. a series of optical images of process (from stage 1 to stage 4) of forming water droplet array based on template-guided method using different size of DFP dots (500 μm (a), and 300 μm (b), respectively).





Figure S7. Response of eight gas microsensors toward different gases under working temperatures at 100, 150, 170, and 250 ℃, and the concentration of the gases is set to 5 ppm.
